# Weight Shame, Social Connection, and Depressive Symptoms in Late Adolescence

**DOI:** 10.3390/ijerph15050891

**Published:** 2018-05-01

**Authors:** Alexandra A. Brewis, Meg Bruening

**Affiliations:** 1 School of Human Evolution and Social Change, Arizona State University, Tempe, AZ 85284-2402, USA; 2 School of Nutrition and Health Promotion, Arizona State University, Phoenix, AZ 85004, USA; meg.bruening@asu.edu

**Keywords:** obesity, weight, adolescents, depression, friendship, peers, stigma, shame, intervention

## Abstract

Child and adolescent obesity is increasingly the focus of interventions, because it predicts serious disease morbidity later in life. However, social environments that permit weight-related stigma and body shame may make weight control and loss more difficult. Rarely do youth obesity interventions address these complexities. Drawing on repeated measures in a large sample (*N* = 1443) of first-year (freshman), campus-resident university students across a nine-month period, we model how weight-related shame predicts depressive symptom levels, how being overweight (assessed by anthropometric measures) shapes that risk, and how social connection (openness to friendship) might mediate/moderate. Body shame directly, clearly, and repeatedly predicts depression symptom levels across the whole school year for all students, but overweight youth have significantly elevated risk. Social connections mediate earlier in the school year, and in all phases moderate, body shame effects on depression. Youth obesity interventions would be well-served recognizing and incorporating the influential roles of social-environmental factors like weight stigma and friendship in program design.

## 1. Introduction

### 1.1. Study Rationale

Childhood and adolescent weight have emerged as a public health priority in many countries because they predict serious morbidities later in life, such as diabetes, coronary heart disease, and a range of cancers [1]. However, there are more immediate negative effects of high body weights of youth related to mental health outcomes, such as depression. For example, one recent meta-analysis determined that adolescents clinically defined as obese had a 40% higher relative risk of depression [2]. A major posited reason is that children’s and adolescent’s high body weights are socially stigmatized, and lead to exposure to multiple forms of weight-related discrimination and mistreatment (such as teasing and rejection) [3,4,5,6,7]. These can emerge even from pre-school (e.g., [8,9,10,11]). Experiencing these forms of social rejection and physical exclusion tend to promote feelings of worthlessness and thus psychological distress in forms relevant to worsening depression or social anxiety [12].

More generally, anti-fat social environments, such as characterize the US and other industrialized nations, tend to promote widespread body shame [13,14]. The chronic belief that one is failing to meet social norms—including related to what is viewed as an acceptable body—is deeply shaming and thus can contribute to depressed mood [15]. Shame is an especially painful powerful, distressing, and—when internalized—also a potentially depressing emotion [16]. This is because it is associated with greater rumination and it also presents more of a direct challenge to highly valued social identities [17]. Importantly for understanding a role for shame in obesity prevention and intervention, this stigma-related psychosocial stress (which manifests in depression symptoms), in itself, then also appears to predict difficulties losing weight or even triggers additional weight gain [3,4,5,18].

While there is growing interest in new ways to understand obesity as a social phenomenon in the context of youth obesity-interventions [19], they rarely address these complexities of social environments or their emotional impacts (focusing rather on individual and family behavior changes to encourage healthy eating and reduce sedentism). There has been a specific call for more studies that identify such social mechanisms relevant to weight in relation to college students in particular, given how understudied they are as a population and the need to better inform college-based interventions [20]. Campus-based healthy-weight interventions, notably, are increasingly deploying weight interventions via social media that connects peers (e.g., [21,22]). This is an additional reason that understanding the role friendship plays in mediating weight stigma and its negative mental health outcomes can assist with more effective intervention design.

### 1.2. Background

There are additional key reasons that representative and/or meaningfully bounded late adolescent US college student communities provide an excellent case to theorize with greater precision how relevant aspects of the social environment interact with weight and associated health conditions like depression. First, anti-fat attitudes and feelings of weight stigma are rife on college campuses [23,24], creating an environment in which the negative effects of weight stigma may be particularly apparent.

Second, entry to college is a critical point for the development of obesity, because relevant lifestyle habits established at this time tend to persist well into adulthood [25]. Moving to campus seems to precipitate rapid weight gain for many [26,27,28]. Studies have varied results, but overall suggest a trend to weight gain in the range of about 10 pounds over time in college [29]. The greatest increases are reported when students first arrive, something in the range of 3–5 pounds in the first year of college has been documented multiple times. For example, Hovell et al. documented the rate of weight increase of new college freshman women as 36 times the speed of matched community women [30]. This is because college-entering adolescents are having to adapt to not just new settings but also newly increased independence [31,32]. The physical changes may include campus foodscapes that include unlimited dining options and easy access to fast food, and with it, shifts in consumption patterns of both food and alcohol that are less healthy [33,34].

Third, when students move to campus for the first time, their social environment is also in massive transition, with new friendships being formed, alongside dealing with homesickness and friendsickness [35], and new routines associated with the first move away from their natal home. Importantly in this regard, an array of studies of campus populations have shown that not all students, however, gain weight; rather, some students exhibit much greater risk of weight gain much more so than others [36]. Meta-analyses also suggest that levels of psychosocial stress might be a key factor explaining why some college students gain and others do not [37], thus linking depression, shame, social connection, and obesity in complex (and likely bi-directional) ways (see [38]).

Our theory building in this study related to the importance of considering the mental health impacts of weight and weight shame, in the contexts of friendships in particular, was based on three sets of literature. First, there is small but growing set of studies from the US showing that those with larger bodies experience lower preference as selection as friends [39], starting in the school yard and extending up through adolescent and adult relationships, including romantic ones (e.g., [40,41]). That is, body weight predicts different friendship opportunities or decisions [42]. Second, a range of social network studies have shown, indirectly, that friend relationships predict convergence of body weight through time [43], even if the exact mechanisms remain very poorly described [44]. Third, perceptions and experiences of social distancing, isolation, loneliness, and rejection (the opposite of friendship) are associated with a greater expression of depression symptoms [45,46].

### 1.3. Study Aim

The aims of this study are to identify effects of body shame on depressive levels of first-year (freshman) university student from the start to the end of the school year, identify if these vary by overweight status, gender, or majority/minority status, and clarify if openness to friendship could mediate or moderate these effects.

## 2. Materials and Methods

### 2.1. Study Design and Setting

The study is based on longitudinal panel study data from 1443 college students living in first-year residence halls at a single university (Arizona State University (ASU)). Freshmen students at ASU are required to live on campus in residence halls unless they have special exemption. Data deployed in this study were collected as part of SPARC (Social impact of Physical Activity and nutRition in College), a large, longitudinal study assessing the nutrition and physical activity choices of students at this very large public university in the US Southwest. The purpose of the SPARC study was to better test how friendship networks impact eating, physical activity, and weight among diverse college freshmen. The SPARC study deployed a socioecological framework that tracked changes in friends’ relationships with diet and exercise and weight status over a single academic year (9 months).

### 2.2. Data Collection, Variables, and Measurement

Study protocols are described fully elsewhere [47], and included repeated collection of detailed behavioral surveys, anthropometric measurements, and ecological momentary assessments of friendship interactions in four phases (1—early fall, 2—late fall, 3—early spring, 4—late spring). Test-retest reliability scores for key variables, assessed from project pilot data, are provided in Bruening et al. [47].

#### 2.2.1. Measurement of Depressive Symptom Levels

Depression symptom levels, the outcome variable, was reported at all four phases. The survey questions were taken from American College Health Association validated protocols [48]. Respondents were asked if in the last month they never (0), rarely (1), sometimes (2), or often (3) (a) felt things were hopeless; (b) were overwhelmed by all you had to do; (c) were very lonely, very sad; (d) so depressed that it was difficult to function. The items responses were summed and averaged to create a score (possible range 1.0–4.0). Scores distributed normally with an expected left skew.

#### 2.2.2. Measurement of Body Shame

The “body shame” variable was derived from a question asked in Phase 1, 3, and 4. The question related to whether respondents strongly disagreed (1), disagreed (2), agreed (3), or strongly agreed (4) that they were embarrassed or ashamed in public due to concerns of being judged because of being overweight; this was from Vartanian and Shaprow’s study [49] in college-age females. For the analysis, we selected a single item related to unwillingness to exercise in public due to be ashamed or embarrassed, being the item the most highly correlated with scores on an anti-fat implicit association test and standard self-report weight stigma scales during pilot validation of the tools (see [24] for details).

#### 2.2.3. Measurement of Openness to Friendship

Social connection was assessed at Phase 1 and Phase 3, and scores reflect “openness to friendship.” The score was the average response to ten statements reflecting openness to friendship. Items were from Buote et al. (2007), Bui (2002), and McAndrew (1998) [32,50,51]. Examples of items are: “Overall, I feel accepted on campus” and “It’s easy for me to make friends on campus.” Response categories differentiated as strongly disagree (1), disagree (2), agree (3), and strongly agree (4), with the friendship score taken as the average response across all ten items (possible range of 1.0 to 4.0). Higher scores reflect greater openness, and were normally distributed.

#### 2.2.4. Measurement of Overweight Status and Weight Concern

“Overweight status” was derived via direct anthropometric measurements, taken to the nearest 0.1 kg by trained assistants using portable Seca scales, and height to the nearest 0.1 cm using portable Seca stadiometers. This was then converted to Body Mass Index measures (BMI), with BMI ≥ 27.5 classified as the cut point for high body weight. Generally, BMI of 30 is used as the cut-point, but as clinical levels of obesity are low in this population (10.3 percent at Phase 1), we made the cut to include the top half of the overweight category as well. Percentage of participants classified as overweight/obese at BMI > 27.5 was 17% in phase 1, 19.5% in Phase 2, 21.2% in Phase 3, and 22.1% in Phase 4. “Weight change” was change in BMI.

#### 2.2.5. Key Covariates

Women are generally observed to be more susceptible than men to both body shaming and depressive/anxious symptomatology [52,53]. Also, the obesity-depression link is generally stronger in women than men [2]; gender was included as a covariate in all the regression analyses (0 female, 1 male). Similarly, ethnicity (particularly majority/white status) has been reported in some (e.g., [54,55]) but not all [56] studies as predicting more body-related concerns and anxieties, so was also included as a key covariate. At all phases, students reported their level of “weight concern,” responding based on whether they felt very underweight, a little underweight, about right, a little overweight, or very overweight. Weight concern is included as a covariate because it has been identified as exerting an independent factor in depression (e.g., [57]). In analyses, this was treated as a binary variable in which respondents were categorized whether they thought they were a little or very overweight (1) versus not (0).

### 2.3. Statistical Analysis

**Figure 1:**
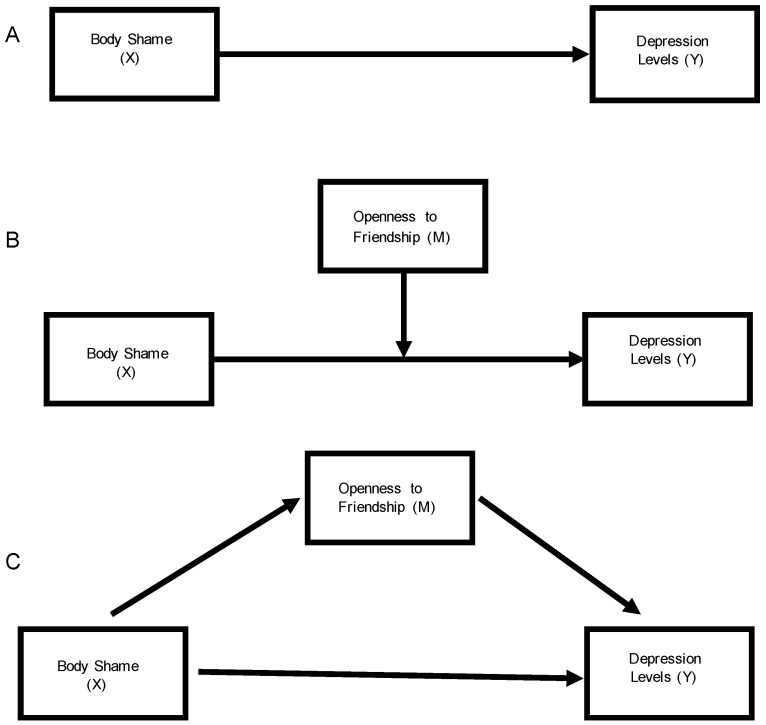
Path diagrams for (**A**) the total effect of body shame on depressive levels; (**B**) the moderated effect of openness to friendship; and (**C**) the mediated effect of body shame on depressive levels through openness to friendship.

Descriptive and regression analyses were done with SPSS v. 22 (IBM Corp., Armonk, NY, USA), with mediation modeling done through SPSS AMOS v.24 (IBM Corp., Chicago, IL, USA) with maximum likelihood estimation. The effect of body shame on the level of depression symptoms was modeled separately for each phase of the school year, including all the covariates in each analysis; however, the relevant weight gain variable was only included in follow-up phases (Phase 2 (end of fall), 3 (start of spring), and 4 (end of academic year)). In addition to the linear regressions that included the covariates, we tested the influence of interactions of friendship with gender and body size on depression levels. The interaction effects were not significant, so were removed from the final models. We then tested the mediation effect of friendship (Figure 1, pathway C) against the direct effect (Figure 1, pathway A), with the sample divided to allow for creation of gender-specific models. As we tested the possible mediation effects of friendship on the relationship of body shame to depression levels in each phase, we then also considered the results in relation to possible moderation effects (Figure 1, pathway B). Because AMOS cannot handle significant missing data and a proportion of students did not complete all four phases, we used Sobel’s test instead of bootstrapping to calculate if the indirect effect was significant in mediation analysis. Alpha for all analyses was set at 0.05.

### 2.4. Ethics Statement

All subjects gave their informed consent for inclusion before they participated in the study. The study was conducted in accordance with the Declaration of Helsinki. Human subjects oversight and approval for this study was provided by Arizona State University IRB (#1309009596).

## 3. Results

### 3.1. Descriptive Statistics

One thousand four hundred forty-three students participated in the first round of data collection at baseline (64.6% female, and 50.7% US-defined minority); 362 students completed all four rounds of data collection (71.5% female, 55.2% US-defined minority). Mean scores and standard deviations for the key variables are provided in Table 1. At baseline, 15 percent of students reported body shame sufficient to want to avoid opportunities for public judgement (15.5% females and 14% males). Those students who were classified as overweight/obese had higher mean levels of body shame (e.g., Phase 1 was 2.1 (±0.95) versus 1.5 (±0.73) for other students, *p* < 0.05). Students who were classified as overweight/obese, but had no body shame (i.e., scored zero on the scale and were “body proud”), had lower mean depression scores (1.88 ± 0.66) compared to others classified as overweight-obese who had body shame (2.53 ± 0.85) (*p* < 0.05).

### 3.2. Linear Regression Analysis

Considering the variables that predicted depression levels (Table 2), greater body shame predicted more depressive symptoms consistently across the school year (*p* < 0.05 at all phases). The associations were statistically strongest in the earlier phases of the school year. Lower friendship scores were a significant direct predictor of higher depressive levels in Phase 1 (when the students were new to campus). Being overweight and being female predicted higher depression levels in Phase 1 only. Level of weight concern was a significant predictor of depression in Phases 2, 3, and 4 once all the other factors were taken into account. Majority ethnicity was not a significant predictor in any phases, once the other variables were taken into account.

### 3.3. Mediation Analysis

The results of the mediation analysis on depression indicates a partial mediating effect of friendship in the earlier phases of the year, whereby the effects of a binary body embarrassment/shame (yes/no) variable on depression were buffered by higher friendship scores (see Table 3 and Table 4). For women students in Phases 1, 2, and 3, those who had more body shame had lower friendship scores, and this then predicts higher depression symptom levels. The same pattern was observed for men in Phases 1 and 2. Fit for all reported models was good (i.e., model *p* < 0.01; root mean square error of approximation (RMSEA) < 0.01; confirmatory fit index (CFI) > 0.93). The significance of the mediation results were confirmed by Sobel’s test (*p* < 0.001).

## 4. Discussion

These results demonstrate that body shame directly, clearly, and repeatedly predicts depressive symptoms levels in a large sample of first year college students tracked across a full school year. Overweight students exhibit more effects, but the effects endure even when weight status is taken into account. This effect is also apparent once other potential covariates such as weight gain, level of weight concern, gender, and majority ethnicity are taken into account.

When we consider the possible mediating/moderating effects of openness to friendship, we find a consistent pattern among both women and men students. The strongest mediating effects are seen earlier in the year, soon after students arrive on campus for the first time. By the final phase of the school year, the effect is not significant as a mediation for either group, although some moderation effect is still apparent. This suggests that early engagement in seeking and maintaining peer friendships in the campus environment is protective against the depressing effects of body shame. This then in turn predicts higher levels of depressive symptoms earlier in the school year. Or rather, both male and female students with greater levels of body shame are less willing to seek and maintain friendships, but the importance of this effect on mood seems to diminish somewhat across the school year. It makes sense that as students integrate into residential life across the school year, the importance of friendship openness to mitigating body shame feelings is diminished, even as the larger influence of body shame on depression remains. 

These findings suggest the benefits of engaging new non-food and non-exercise based interventions at the times students first arrive on campus that could help adolescents manage weight. From a practical perspective, it suggests that widely applied campus-based efforts to encourage positive body image (such as Body Project [58]) that are most often concerned with preventing eating disorders, might also be able to buffer new students’ risk of both weight gain and depression as well. In addition, importantly, this study shows that men students are also vulnerable to the enculturated effects of body shame on depressive symptom risk while new to campus, so interventions should target both genders. It also reinforces the suggestion that shifting health promotion strategies that emphasize total wellness and body self-esteem, and understand that weight and weight gain are shaped by the social environment as well as the physical one, could be ultimately more effective for helping first year students better manage their weight. Other recommendations for reducing body shame and stigma include: youth-targeted media, provider training, and (importantly for universities) creating policies that address and prohibit weight discrimination (see [19]). 

The findings, drawn from a relatively large sample of late adolescents (college students) bounded in a meaningful community (residence halls), also provide another important observation: weight-interventions, broadly conceived, are not just relevant to those with higher body weights. While students with larger bodies experience more body shame and more depression, a significant percentage of all students report feelings of shame to a level that makes them want to avoid public notice related to their bodies. In addition, although women students showed a stronger effect at baseline, once living in the university environment. this effect similarly impacted both genders. In addition, when modeled, we observe that this then results in higher levels of both depression symptoms for females and males alike. 

While these mental health effects of high body weights are often overlooked in obesity intervention design, it is important to recognize that common mental illnesses are co-morbidities with weight as much as the chronic physical illnesses such as diabetes. Reducing these effects in students of all body sizes is relevant to obesity interventions, because depression is an established risk factor for subsequent weight gain and, hence, the later adult emergence of obesity [2]. 

The study results also suggest that the growing emphasis on social media to support adolescent weight management must understand that friendship connections related to weight and weight issues can be beneficial, but also stressful. Particularly, with both greater body shame and higher body weights, openness to engage in social connection can lessen, as is seen in this sample. This needs to be taken into account in intervention designs. 

## 5. Limitations

A major strength of the study is the large sample size, with repeat observations made across nine months. The study limitations include the limited number of survey questions used as the basis to define some key variables. Our measure of body shame was drawn from just two questions, and not from a validated scale. This study only considers how body shame predicts depression through the influence of friendship; there are theorized counter-directional relationships (e.g., depression shapes friendship and body shame) [3] not considered here within this study design. Further, the longitudinal data is only available for 25% of the total sample, and biases about who was retained in the study may mean those who were measured at all four time periods may mean the results do not exactly represent the total sample. 

## 6. Conclusions

The first year of university has long been identified as one in which weight gain is especially accelerated, at least for a sub-set of students. Here, we show that overweight/obese students of both genders who are body ashamed are less likely to seek or maintain friendships, and this then additionally heightens their risk of depressive symptoms. The effects are most pronounced when students first arrive on campus, but the moderating effects remain even as they integrate into campus life. Weight interventions in late adolescence will benefit from recognizing weight as gained and lost within and through the influence of social as well as physical environments and that weight has negative mental as well as physical health impacts. The mental health effects of high body weight are arguably more immediate and pressing in the context of university life, because they not only predict subsequent weight gain, but also such factors as retention and graduation. That students are most susceptible to these mental health effects when they first arrive on campus is especially relevant to retention efforts. Effective campus-based strategies to tackle being overweight should recognize and incorporate this within their design, including emphases on body positivity. Addressing weight related stigma in adolescent interventions may have other less-expected general health benefits too; reducing psychosocial stress could even reduce other varied forms of risk-taking relevant to weight control, like binge drinking [59].

## Figures and Tables

**Table 1:** Mean descriptives by phase.

Key Variables of Interest	Phase 1 Start of Fall Semester	Phase 2 End of Fall Semester	Phase 3 Start of Spring Semester	Phase 4 End of the Spring Semester
	*n* = 1430 64.9% Female	*n* = 629 70.5% Female	*n* = 555 71.2% Female	*n* = 534 70.5% Female
	Means (SD)			
Depression levels (0–20 scale)	5.34 (3.8)	5.85 (3.9)	5.13 (3.8)	5.82 (4.0)
Friendship scores (1–4 scale)	3.3 (0.4)	--*	3.1 (0.4)	--*
Body shame (0–4 scale)	1.61 (0.8)	1.61 (0.8)	--*	1.66 (0.8)
Body mass index (BMI)	24.1 (4.9)	24.6 (5.1)	24.6 (5.1)	29.4 (5.3)
Weight concern (believes is overweight)	3.3 (0.9)	3.5 (0.8)	3.5 (0.9)	--*
Weight change since Phase 1 (based on BMI change)	--	.69 (6.9)	0.72 (7.3)	0.63 (7.2)

* Used prior phase values in models.

**Table 2:** Regression predicting levels of depressive symptoms across four study phases.

Outcome	Predictor	B	SE	95% CI Lower	95% CI Upper	*p*
Depression at start of school year (*N* = 1443)	Body shame	0.305	0.025	0.256	0.354	0.000
	Friendship score	−0.363	0.044	−0.449	−0.276	0.000
	Overweight status	−0.133	0.058	−0.246	−0.020	0.021
	Believes is overweight	0.005	0.027	−0.047	0.058	0.846
	Male gender	−0.193	0.041	−0.273	−0.112	0.000
	Majority ethnicity	0.056	0.037	−0.018	0.129	0.137
Depression at end of fall semester (*n* = 629)	Body shame	0.240	0.064	0.114	0.366	0.000
	Friendship score	−0.114	0.114	−0.339	0.111	0.320
	Overweight status	−0.055	0.138	−0.326	0.215	0.687
	Believes is overweight	0.170	0.066	0.040	0.300	0.011
	Male gender	−0.003	0.100	−0.200	0.194	0.978
	Majority ethnicity	0.098	0.090	−0.079	0.275	0.276
	Weight change to date	0.001	0.009	−0.016	0.018	0.922
Depression at start of spring semester (*n* = 555)	Body shame	0.172	0.076	0.023	0.322	0.024
	Friendship score	−0.219	0.143	−0.501	0.062	0.126
	Overweight status	−0.022	0.173	−0.364	0.320	0.900
	Believes is overweight	0.204	0.069	0.069	0.340	0.003
	Male gender	−0.093	0.111	−0.313	0.126	0.403
	Majority ethnicity	0.189	0.103	−0.014	0.392	0.068
	Weight change to date	0.010	0.008	−0.006	0.026	0.220
Depression at end of school year (*n* = 534)	Body shame	0.287	0.085	0.119	0.455	0.001
	Friendship score	−0.109	0.160	−0.426	0.208	0.497
	Overweight status	−0.049	0.183	−0.411	0.312	0.788
	Believes is overweight	0.241	0.091	0.061	0.420	0.009
	Male gender	0.188	0.143	−0.094	0.469	0.190
	Majority ethnicity	0.237	0.123	−0.007	0.481	0.056
	Weight change to date	0.012	0.011	−0.010	0.035	0.284

**Table 3:** Mediation analysis examining friendship on the relationship of body shame to women students’ depression levels (*n* = 927). *** *p* < 0.001.

Phase	Predictor	Outcome	Estimate (b)	SE	*p*
Start of the school year	Body shame	Friendship	−0.110	0.016	***
	Friendship	Depression	−0.509	0.055	***
	Body shame	Depression	0.291	0.028	***
End of fall semester	Body shame	Friendship	−0.100	0.022	***
	Friendship	Depression	−0.266	0.085	0.002
	Body shame	Depression	0.262	0.042	***
Start of spring semester	Body shame	Friendship	−0.059	0.026	0.022
	Friendship	Depression	−0.444	0.090	***
	Body shame	Depression	0.324	0.045	***
End of spring semester	Body shame	Friendship	−0.033	0.027	0.210
	Friendship	Depression	−0.227	0.104	0.029
	Body shame	Depression	0.346	0.043	***

**Table 4:** Mediation analysis examining friendship on the relationship of body shame to men students’ depression levels (*n* = 508). *** *p* < 0.001.

Phase	Predictor	Outcome	Estimate (b)	SE	*p*
Start of the school year	Body shame	Friendship	−0.066	0.027	0.013
	Friendship	Depression	−0.155	0.069	0.025
	Body shame	Depression	0.285	0.041	***
End of fall semester	Body shame	Friendship	−0.099	0.046	0.031
	Friendship	Depression	−0.133	0.042	0.002
	Body shame	Depression	0.269	0.095	0.005
Start of spring semester	Body shame	Friendship	−0.133	0.042	0.002
	Friendship	Depression	−0.145	0.173	0.402
	Body shame	Depression	0.269	0.095	0.005
End of spring semester	Body shame	Friendship	−0.133	0.043	0.002
	Friendship	Depression	−0.234	0.202	0.245
	Body shame	Depression	0.250	0.092	0.007
